# Depth Perception Not Found in Human Observers for Static or Dynamic Anti-Correlated Random Dot Stereograms

**DOI:** 10.1371/journal.pone.0084087

**Published:** 2014-01-08

**Authors:** Paul B. Hibbard, Kenneth C. Scott-Brown, Emma C. Haigh, Melanie Adrain

**Affiliations:** 1 School of Psychology and Neuroscience, University of St Andrews, St Andrews, Fife, United Kingdom; 2 Department of Psychology, University of Essex, Colchester, Essex, United Kingdom; 3 Centre for Psychology, School of Social and Health Sciences, Abertay University, Dundee, United Kingdom; 4 Scottish Mental Health Research Network, Royal Edinburgh Hospital, Edinburgh, United Kingdom; Université Paris 5, and CNRS, France

## Abstract

One of the greatest challenges in visual neuroscience is that of linking neural activity with perceptual experience. In the case of binocular depth perception, important insights have been achieved through comparing neural responses and the perception of depth, for carefully selected stimuli. One of the most important types of stimulus that has been used here is the anti-correlated random dot stereogram (ACRDS). In these stimuli, the contrast polarity of one half of a stereoscopic image is reversed. While neurons in cortical area V1 respond reliably to the binocular disparities in ACRDS, they do not create a sensation of depth. This discrepancy has been used to argue that depth perception must rely on neural activity elsewhere in the brain. Currently, the psychophysical results on which this argument rests are not clear-cut. While it is generally assumed that ACRDS do not support the perception of depth, some studies have reported that some people, some of the time, perceive depth in some types of these stimuli. Given the importance of these results for understanding the neural correlates of stereopsis, we studied depth perception in ACRDS using a large number of observers, in order to provide an unambiguous conclusion about the extent to which these stimuli support the perception of depth. We presented observers with random dot stereograms in which correlated dots were presented in a surrounding annulus and correlated or anti-correlated dots were presented in a central circular region. While observers could reliably report the depth of the central region for correlated stimuli, we found no evidence for depth perception in static or dynamic anti-correlated stimuli. Confidence ratings for stereoscopic perception were uniformly low for anti-correlated stimuli, but showed normal variation with disparity for correlated stimuli. These results establish that the inability of observers to perceive depth in ACRDS is a robust phenomenon.

## Introduction

### Binocular depth perception and the correspondence problem

Differences in the images formed in our two eyes provide valuable information about depth. To make use of this information, we need to determine the differences in location of corresponding points in the two images. This process depends on computations akin to the calculation of a cross-correlation between local samples from the two images [1]–[5] with the initial stages of this computation occurring in cortical area V1 [6]–[8].

An important stimulus in the development of our understanding of binocular stereopsis is the random dot stereogram, or RDS [9]. The image presented to one eye in a typical RDS consists of a collection of randomly located bright and dark dots. In the image presented to the other eye, a subset of these dots is shifted, so as to produce a binocular disparity. Under the right conditions, observers will then see depth appropriate for the introduced disparities. This shows that people are able to see depth purely on the basis of binocular disparity, in the absence of any other cues to the depth perceived.

### Anti-correlated stereograms and the perception of depth

Julesz also introduced a variant of the RDS called the anti-correlated random dot stereogram (ACRDS) [9]. Here, the luminance polarity of each element of the stereogram is reversed. Bright elements in the left eye's image are thus presented as dark elements in the right eye's image, and *vice versa.* Julesz showed that observers are able to perceive depth in correlated RDS (CRDS), but not ACRDS [9].

Earlier research has however established that depth can be seen in some stimuli in which the polarity is reversed between the two eyes. Helmholtz showed that stereoscopic depth could be seen in a simple geometric figure [10]. Treisman showed that a reversed polarity diagram of a ring, but not a disc, supported stereoscopic depth [11]. One explanation of this difference is that observers are able to match luminance edges with the same sign of gradient [12]. With a thin object, there are two gradients of opposite signs in close proximity at each if its edges. For objects in which the light-dark and dark-light transitions are more separated in space, there may be no edge of the correct polarity that is close enough across the two images to be matched. This same-sign matching rule can account for the fact that depth might be reversed, compared to that predicted from a simple consideration of the disparities present, when the polarity of some elements is switched [13]–[14]. Observers were also unable to perceive depth in reversed polarity stereograms containing letters [15]. Julesz proposed that the key difference between ACRDS, and those studies in which depth can be seen when polarity is reversed, is the spatial density of contours in the images [9].

Other studies using ACRDS have produced rather mixed results. Cogan et al. asked observers to rate the quality of depth in CRDS and ACRDS, as a function of their density, and the stimulus onset asynchrony (SOA) between the presentation of the left and right eyes' images [16]. During the time that the random dot stimulus was presented to one eye, the other eye was presented with a uniform grey field. For CRDS, the quality of depth was high for all densities, and short SOAs, but tended to decrease for SOAs beyond around 60 ms. For ACRDS, the quality of depth tended to reduce for short SOAs, particularly when the density was high. Thus, with short SOAs, high quality depth tended only to be seen for low element densities; the density at which depth deteriorated varied between their three observers. Their observers also varied in the quality of depth perceived, with one never reporting high quality depth perception for ACRDS, and showing almost no improvement as the dot density was reduced.

Cumming et al presented observers with ACRDS of a square against a zero-disparity background, with both the square and the surround anti-correlated [17]. They were unable to measure thresholds even with very low densities (1%). Only after extensive training, with feedback, were their observers able to discriminate the sign of disparity reliably. Even after training, performance remained at chance when the dot density was too high.

Read and Eagle created stereograms from one-dimensional vertical noise patterns [18]. When these patterns were broadband, some of their observers reported reversed depth in anti-correlated stimuli, while others responded at chance. They found similar results with two-dimensional noise patterns: three of their observers (including the two authors) showed a weak but reliable tendency to report reversed depth in anti-correlated stereograms. The other two observers responded at chance. With ACRDS, reversed depth was again reported by two out of four observers. However, when the anti-correlated target was presented against a background that was also anti-correlated, none responded better than chance. Observers were however able to discriminate the sign of disparity correctly for correlated targets against an uncorrelated background.

Tanabe et al used RDS consisting of a correlated or uncorrelated central target area, surrounded by a correlated annulus [19]. Stimuli were presented with an interocular delay that varied between 12 and 156 ms. For short delays, there was a tendency for observers to report reversed depth with anti-correlated stimuli. As the delay increased, performance rose to chance; depth was perceived in the correct direction for delays of greater than 60 ms, before falling back to chance when the delay was 160 ms. There were however significant individual differences in the reliability with which their observers reported reversed depth for anti-correlated targets with short interocular delays. In a second experiment, no observers were able to make fine shape judgements (reporting the orientation of a T-shape defined by a disparity) in anti-correlated stimuli. The main difference between the stimuli used in this study and those used by others is the presence of a correlated background. When both the centre and surround were anti-correlated, responses were at chance. Doi et al. reported reversed depth for anti-correlated stimuli with a large disparity (28.8 arc min), but chance performance for stimuli with a small disparity (1.8 arc min) [20]. Reversed depth was reported for presentation times of both 1.5s and 94 ms.

In summary, when RDS contain both an anti-correlated background and an anti-correlated target, observers are unable to discriminate the disparity of the target. When an anti-correlated target is presented without a background, or against a correlated background, then some of the people, some of the time, perceive depth in the reversed direction.

### Linking depth perception with physiological responses

The questions of whether and when observers can see depth in ACRDS have proved critical in the assessment of theoretical models of stereopsis [21] and have played a central role in understanding how neural responses to binocular information relate to the perception of depth. Binocular neurons in V1 respond to ACRDS, albeit with a reduced magnitude, and an inversion of their disparity tuning function, in comparison with their responses to CRDS [22]. This means that the largest response to ACRDS is found for stimuli with a disparity giving the smallest response to CRDS. The standard energy model of V1 neurons [3] predicts this inversion in the disparity tuning function, and modifications of the model have been proposed that also account for the reduced magnitude of response [21]. It has been argued that this inversion of the disparity tuning function can account for the reversal in perceived depth found in some studies [18]–[20]. However, a comparison between V1 responses and psychophysical results is often taken as direct evidence that responses in binocularly tuned V1 neurons are not sufficient for the perception of depth [6–7], [22]–[23]. This is because, it has been argued, while V1 neurons reliably respond to changes in disparity in ACRDS, human and macaque observers do not. This has led to the use of ACRDS in investigations using single cell recordings in the macaque and fMRI in humans that have sought to determine whether the responses in other, extrastriate visual areas are more closely tied to the perception of depth. Disparity-selective responses to ACRDS similar to those in V1 are found in V2 [24]. In the ventral stream, disparity-selective responses to ACRDS are not found in areas V4 [25] or TEs [26]. In the dorsal stream, responses to ACRDS are intermediate to those of V1 and V4 [27]. fMRI in humans has shown disparity-selective responses to ACRDS in early visual areas and intermediate ventral areas, but not the higher ventral stream area LO, or dorsal area hMT+/V5 [28].

The currently equivocal answer to the question of whether people can perceive depth in some types of ACRDS needs to be clarified if were are to firmly establish our understanding of the link between depth perception and the disparity-tuned responses of binocular cortical neurons.

### Aims of the current study

The current study sought to provide a clearer answer than currently exists to the question of whether people can indeed see depth, reversed or otherwise, in ACRDS. Our first goal was to use a much larger sample of observers than has been tested before. Previous studies of slant perception [29], motion in depth [30], and relative depth intervals [31] have demonstrated the need to study individual differences in order to fully understand the underlying mechanisms of binocular depth perception [32]. Here, we use this approach to determine the extent to which reliable disparity discrimination can be found for ACRDS presented with a correlated background. All previous studies have found that observers *cannot* discriminate disparity in ACRDS in which the background is also anti-correlated. Three studies have found that observers can perceive depth when there is no background, or the background is correlated [18]–[20]. Even in these three studies, disparity discrimination was at best unreliable, and not found for all observers.

Our second goal was to determine whether responses are affected by the stimulus presentation time. This manipulation was included following the suggestion that perceived depth for reversed polarity stimuli might depend on the transient channel for stereopsis [33]. While Pope et al [33] argued that this channel would not support apparent depth in ACRDS, since it depends on low spatial frequency components, the manipulation was included to rule out one possible reason for failing to find reliable disparity discrimination.

Our third goal was to record our observers' confidence in their responses. Since better-than-chance disparity discrimination was not shown by all observers in previous studies, and tended to be unreliable, we reasoned that this discrimination was unlikely to be accompanied by a vivid sensation of stereoscopic depth. Reliable responses might be possible in the absence of stereopsis. To investigate this possibility, we introduced a ‘commentary key’, as used in the study of blindsight [34]–[35], to allow our observers to report their confidence in their forced-choice responses. It was predicted that confidence would reflect the reliability of responses for correlated stereograms. In contrast, if observers were able to respond to disparity in the absence of an experience of depth for ACRDS, confidence might remain low even if responses were above chance.

## Materials and Methods

### Ethics statement

All procedures were approved by the University of St Andrews University Teaching and Research Ethics Committee. Participants provided written informed consent to participate in the study.

### Experiment one

#### Apparatus

Stimuli were presented on a 21inch Sony Trinitron CRT monitor running at 100 Hz. The luminance response of the monitor was measured using a Minolta LS-110 luminance meter. CrystalEyes LCD shutter goggles were used to achieve stereoscopic presentation. To minimise cross-talk in the goggles, only the red phosphor of the monitor was used. Stimuli were created and presented using MATLAB and the Psychophysics Toolbox extensions [36]–[37]. Stimuli were viewed from a distance of 115 cm, in a dimly lit laboratory.

#### Stimuli

**Figure 1:**
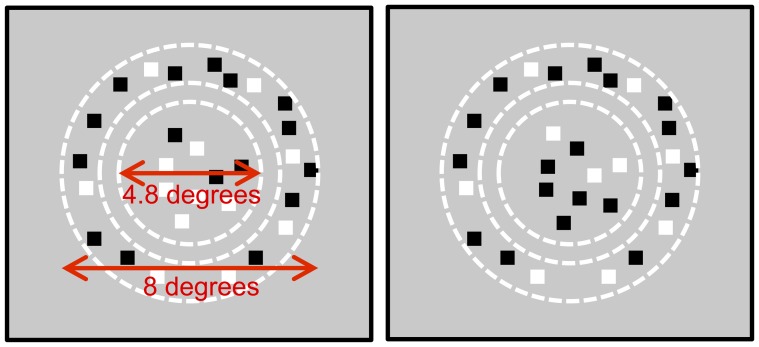
Schematic illustration of the stimuli used. The two squares show the left and right eyes' images. Randomly positioned bright and dark dots were presented in an outer annlus and an inner circle. The dots in the outer annlus were always correlated, having the same polarity in the two eyes' images. The dots in the central circle were either correlated or, as shown here, anticorrelated. The dots in this region were were given a disparity between the two eyes' views.

The stimuli were random dot stereograms, comprising dark (1.8 cdm^−2^) and bright (20.6 cdm^−2^) red dots presented against a red background (11.3 cdm^−2^). Each dot was a 0.14° square. The dot density was 25%; half the dots were dark, and half were bright. Each stereogram consisted of dots presented in a central circular region with a diameter of 4.8° and a surrounding annulus, with a larger diameter of 8° and a smaller diameter of 5.5°. An illustration of the stimuli is given in figure 1.

The dots in the annulus were always correlated, and presented with zero disparity. Within a block of trials, the dots in the circle were either always correlated, or always anti-correlated. These dots were presented at nine disparities (0, ±4, ±10, ±16 and ±20 arc min). These values were chosen to span a range of disparities that are likely to support clear stereopsis, and were not intended to be used to measure a disparity threshold; Read and Eagle [18] argued that some previous studies (e.g. [17]) might not have demonstrated depth perception with ACRDS because the disparities used were too small. 20 stimuli of each disparity were presented within each block, in a different randomised order for each observer. Four presentation durations were used (80, 120, 200 and 400 ms), and this was kept constant within a block. Observers therefore completed 8 blocks (four presentation times, for correlated and anti-correlated stimuli), with 180 trials in each block.

#### Procedure

The order of presentation of the eight blocks of trials was determined for each observer using a Latin Square design. Each block began with the presentation of a small, central fixation cross with a luminance of 1.8 cdm^−2^ against a red background with a luminance of 11.3 cdm^−2^. The observer initiated the presentation of the trials by pressing a response key on the keyboard. After the presentation of each trial, the fixation cross was again presented, and the observer was asked to indicate, using one of two response keys, whether the dots in the central circular region appeared closer or further away than those in the surrounding annulus. The next trial was presented once the observer had made their response. After all 8 blocks had been presented, observers were asked to judge their confidence in their ratings. This was done by presenting all 8 blocks again, but in this case with just one presentation of each stimulus. Rather than judging the depth of the stimulus, the observers were asked to rate how confident they were that they saw near or far depth. This was judged on a 7 point scale (1 =  “very confident”, 7 =  “not at all confident”).

#### Observers

37 observers participated in the study (27 female, 10 male). The age range of the observers was 18–40. Observers were all staff or students from the University of St Andrews.

### Experiment two

The apparatus, methods and procedure were the same as in experiment one, except for the following differences. First, only a presentation duration of 80 ms was used. Second, both static and dynamic stimuli were used, for both CRDS and ACRDS. For the dynamic stimuli, a new random dot pattern was created for each frame. Third, 40 trials were completed for each disparity. Finally, no confidence ratings were collected. This experiment was completed by three observers, including the author PBH.

## Results and Discussion

### Average accuracy of responses

**Figure 2:**
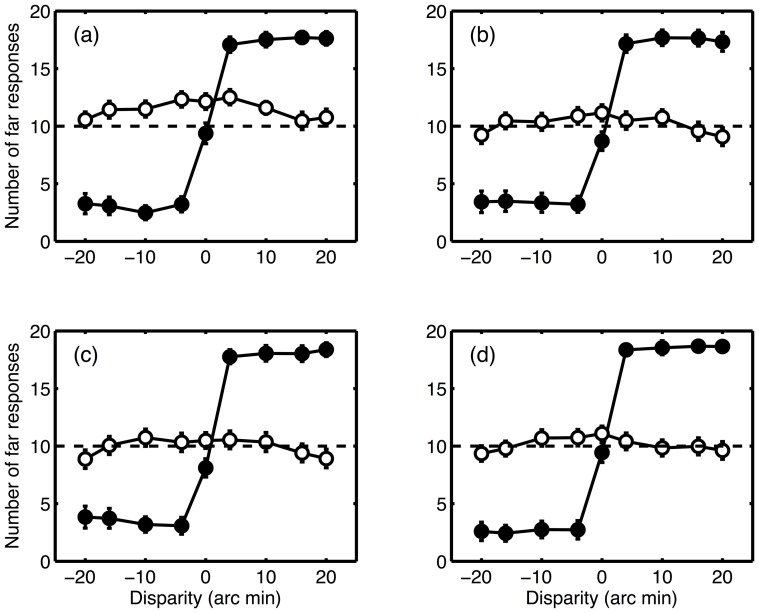
Mean number of far responses (out of 20) as a function of disparity. Negative values represent crossed disparities, postive values uncrossed disparities. Filled symbols show the results for CRDS (•), unfilled symbols for ACRDS (○). Results for the four presentation times are plotted separately ((a) 80 ms; (b) 120 ms; (c) 200 ms; (d) 400 ms). In each case, the dashed line shows chance performance, and error bars show ±1 standard error of the mean.

Figure 2 shows the mean number of “far” judgements for experiment one, across observers, as a function of disparity. Results are plotted separately for each presentation time. Crossed disparities (near depth) are plotted as negative values, and uncrossed disparities (far depth) as positive values. For CRDS, observers made mainly “near” responses for crossed disparities, mainly “far” responses for uncrossed disparities, and approximately equal numbers of each when the disparity was zero. The results were very similar for all four presentation times. The data were analysed using a two-way (disparity-by-presentation time) repeated measures ANOVA. Where significant deviations from sphericity were detected, the degrees of freedom of this test were adjusted using a Greenhouse-Geisser correction. There was a main effect of disparity (F(1.54,55.5) = 139.2; p<0.001; partial η^2^ = 0.795), but no effect of presentation time (F(3,108) = 0.508; NS; partial η^2^ = 0.014). There was also a significant interaction (F(6.82,245) = 2.206; p<0.005; partial η^2^ = 0.058). This reflects the fact that performance tended to improve slightly as the presentation time increased. This improvement would not be expected to affect the mean number of responses in a particular direction, as these would always be expected to be balanced between ‘near’ and ‘far’, but would be expected to change the way in which these depend on disparity.

The results were very different for ACRDS. Observers made approximately equal numbers of “near” and “far” responses for large magnitudes of disparity (regardless of sign) and showed a slight tendency to report the target as further than the surround when disparity was small or zero, as noted previously by Cogan et al. [16]. Results were not affected by the presentation time. Repeated measures ANOVA showed a significant effect of disparity (F(4.04,145) = 10.1; p<0.001; partial η^2^ = 0.457), no effect of duration (F(2.27,81.8) = 1.93; NS; partial η^2^ = 0.013), and no significant interaction (F(12.0,432) = 0.73; NS; partial η^2^ = 0.282).

**Figure 3:**
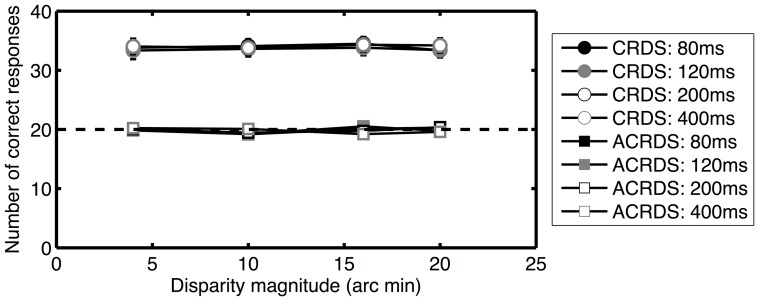
Mean number of correct responses, as a function of the magnitude of disparity. CRDS results are plotted as circles, ACRDS results are plotted as squares. The legend indicates the symbols used for each stimulus type and presentation time. The dashed line shows chance performance, and error bars show ±1 standard error of the mean.

The important finding here is that, although responses were affected by the magnitude of disparity for ACRDS, they were not affected by its sign. That is, observers were not able to discriminate between stimuli with crossed and uncrossed disparities. To illustrate this more clearly, the accuracy of results for stimuli with non-zero disparities is presented in figure 3. Here, results for crossed and uncrossed responses are combined, and the number of correct responses (out of 40) is plotted as a function of the magnitude of disparity. Observers on average made mostly correct responses for CRDS, but responded at chance for ACRDS.

### Individual differences

One reason for using a larger sample of observers than previous studies is that those studies appeared to show individual differences [16]–[19]; while some observers reported depth in ACRDS, others did not. We therefore assessed whether there were differences in the reliability with which our observers responded. It is possible, for example, that a subset of observers responded better (or worse) than chance to the ACRDS, but that this is masked in the average results by the chance-level responses of other observers. To determine whether observers were able to use disparity in order to make correct depth judgements, for each stimulus type the total number of correct responses (out of 640) across all durations, and all non-zero disparities, was calculated. The probability of obtaining at least this number of correct responses by chance was calculated according to a binomial distribution. For CRDS, the probability calculated was less than 5% in all but one case. For ACRDS, a probability of less than 5% was found for only one observer. Given the number of sets of data analysed (37) this is no more than expected by chance. In summary, all but one of our observers were able to discriminate depth from disparity for CRDS, but we found no evidence that any could do this for ACRDS.

### Confidence ratings

**Figure 4:**
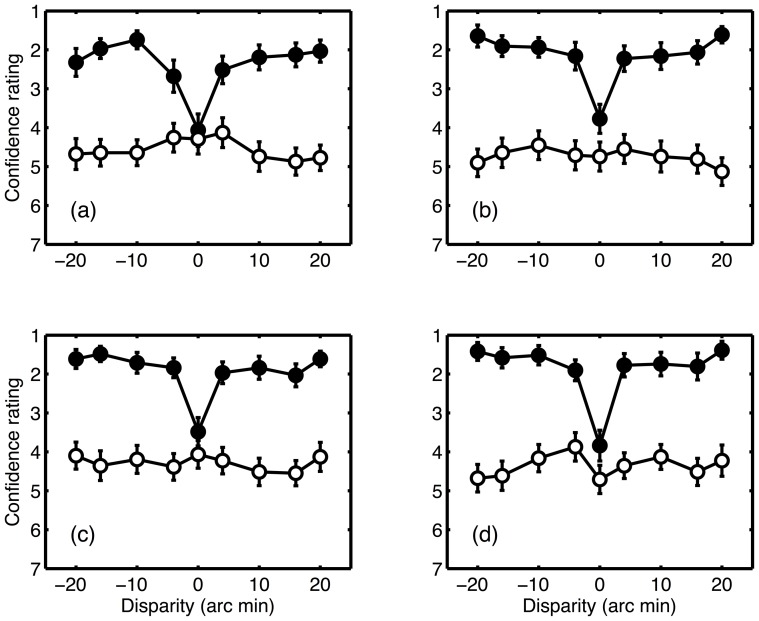
Mean confidence ratings as a function of disparity. Filled symbols show the results for CRDS (•), unfilled symbols for ACRDS (○). Results for the four presentation times are plotted separately ((a) 80 ms; (b) 120 ms; (c) 200 ms; (d) 400 ms). Small values represent high confidence, and large values low confidence. Error bars show ±1 standard error of the mean.

Average confidence ratings are presented in figure 4. Observers had high confidence in their judgements for CRDS with a non-zero disparity, and low confidence for stimuli with a zero disparity. This is as expected; with zero disparity there is no correct answer, so observers will presumably have been guessing when making depth judgements. Confidence ratings were uniformly low for ACRDS. A three-way repeated measures ANOVA showed significant effects of correlation (CRDS versus ACRDS) (F(1,30) = 52.4; p<0.001; partial η^2^ = 0.636), duration (F(3,90) = 5.54; p<0.005; partial η^2^ = 0.330) and disparity (F(4.43, 133) = 14.8; p<0.001; partial η^2^ = 0.156). There was also a significant interaction between correlation and disparity (F(4.14,124) = 13.0; p<0.001; partial η^2^ = 0.303). Confidence was greater for ACRDS than for CRDS, and at longer durations. Separate duration-by-disparity ANOVAs were carried out for ACRDS and CRDS, to explore the significant interaction. For CRDS, there were significant main effects of duration (F(1.97,59.1) = 4.89; p<0.005; partial η^2^ = 0.140) and disparity (F(2.71,81.5) = 21.35, p<0.001; partial η^2^ = 0.416) but no significant interaction. For ACRDS, there were no significant effects. In summary, for CRDS confidence was low when disparity was zero, and increased with increasing presentation time. For ACRDS, confidence was uniformly low.

While our stimuli were modelled on those used by Tanabe et al. [19], there were two important differences. Tanabe et al. [19] argue that studies typically do not provide a fair comparison of depth perception in CRDS and ACRDS, since the latter have a lustrous appearance, while the former do not. They raised the possibility that observers might not attempt to discriminate depth in the absence of a clear, crisp depth percept. In their experiment, they used dynamic stimuli, in which a new random dot pattern was generated on every frame. They also used an inter-ocular delay between the presentation of the stimulus between the two eyes, using a broad range of delays. Since this delay will also reduce the crispness of the subsequent depth percept, the difference between CRDS and ACRDS will be reduced. However, even with this manipulation, the perception of depth in ACRDS was modest. Moreover, two other studies did not use an interocular delay [18]–[20], and both reported reliable reversed depth in ACRDS, so this manipulation cannot be critical for reliable discrimination of disparity. Reversed depth in ACRDS was also found for static stimuli by Read and Eagle [18]. However, to determine whether the use of static stimuli was an important factor in the current study, a second experiment was run in which both static and dynamic stimuli were used.

**Figure 5:**
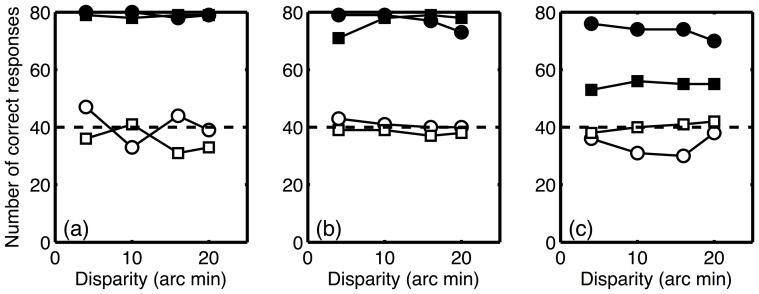
Number of correct responses (out of 80), as a function of disparity for static CRDS (•), dynamic CRDS (▪), static ACRDS (○) and dynamic ACRDS (□). Results are plotted separately for (a) author PBH and (b) and (c) two naïve participants. In each case, the dashed line shows chance performance.

The results of the second experiment are plotted in figure 5. The use of static, rather than dynamic, stimuli did not determine whether observers could reliably discriminate depth for either stimulus type. All observers were able to discriminate depth from disparity for CRDS, but not for ACRDS.

We found no evidence that observers were able to discriminate the sign of disparity for anti-correlated targets, when presented against a correlated background. Consistent with this, confidence ratings were uniformly low for anti-correlated stimuli, but showed normal variation with disparity for correlated stimuli. Our results provide clear evidence that these stimuli did not support the perception of depth, in observers who showed reliable discrimination for correlated stimuli.

While our results, taken alone, provide an unambiguous answer, they are at variance with some previous reports [18]–[20]. Indeed, one of our goals was to establish the extent of reversed depth perception in ACRDS, which these previous studies have found to be unreliable and idiosyncratic. Against our expectations, using a much larger sample of observers we found no evidence for perception of reversed depth in ACRDS.

An important difference between our experiment and previous studies is the number of repetitions used. Since we wanted to assess a large number of observers, it was important to keep the duration of the experiment as short as possible. We used 40 repetitions for each magnitude of disparity, whereas Read and Eagle used 80, Doi et al. used 60 and Tanabe et al. used at least 60. This reduction in the number of trials reduced that statistical power of our study to detect performance that was significantly different from chance. This could potentially explain why we did not find any evidence that observers could discriminate disparity in ACRDS. To assess this possibility, we calculated whether the results of previous studies would still have been significant given a smaller number of trials. For the assessment of Tanabe et al.'s results, we calculated the binomial probability that the proportion of correct responses for anti-correlated stimuli with the minimum interocular delay would represent a significant deviation from chance, had only 40 trials been run. The result for their fourth observer was inferred from the average over the participants. For Doi et al.'s data, we did the same for the results for ACRDS with a coarse disparity. We calculated that significant reversed depth (p<0.05 using a binomial test) would have been detected with 40 trials for three of Tanabe et al.'s observers. Significant reversed depth would have been detected for all of Doi et al.'s observers.

For Read and Eagle's data, a different approach was taken since, as in our study, they presented a range of disparities to their observers. This allowed us to use the same analysis on their data (assuming that only 40 trials had been run) as we had used on ours. We calculated the probability that these results could have been obtained by chance for the two observers (JR and RAE) who demonstrated reversed depth perception. Using our analysis, and assuming 40 repetitions, these results would have been significantly different from chance (JR: p<10^−10^; RAE p<10^−6^).

**Figure 6:**
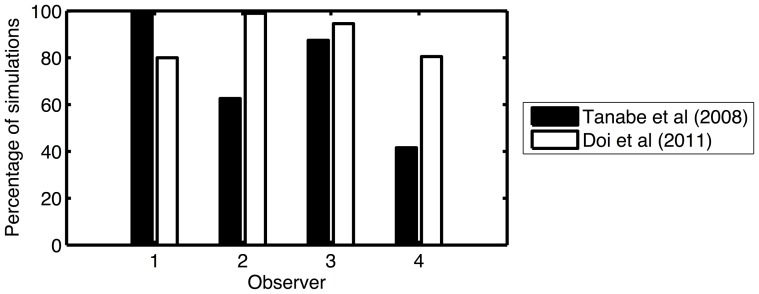
The percentage of the 10000 simulated experiments for which a simulated observer, based on the data of two published studies, would have produced a result significantly different from chance.

To assess this issue in more detail, we simulated the results of each experiment, based on the actual number of correct responses reported, over 10000 runs. In each of these runs, we simulated 40 trials for each stimulus, with the probability of a correct response taken from the empirical data in each study. From these results, we calculated the proportion of runs on which a significant (p<0.05) deviation from chance was found, for each simulated observer. The results for the simulations of Tanabe et al.'s and Doi et al.'s studies are presented in figure 6. For the simulation of Read and Eagle's experiment, a significant departure from chance performance was obtained on all 10000 simulations of JR and RAE's performance. Given that this simulation used the same statistical analysis as used in the current study, we can be confident that we would have been able to detect the perception of reversed depth using our design for at least some observers.

The use of static rather than dynamic stimuli also did not appear to have been responsible for the lack of reversed depth for anti-correlated stimuli. The temporal dynamics of the stimuli are important for a number of possible reasons. Firstly, the biphasic temporal response function of cortical neurons [38] has been used to account for the perception of depth in the correct direction when a suitable delay between the presentation of the left and right eye's images is present [19]. Given the small delay introduced by the stereogoggles (10 ms) we predicted that, if depth had been perceived, it would have been in the reversed direction [19]. Secondly, the perception of depth in anti-correlated stimuli has been associated with processing in the transient system [33]. This system depends on the presence of information at high temporal frequencies. Pope et al. [33] argued that the spatial frequency tuning of the transient system makes it unsuitable for the detection of disparity in random dot stereograms. Here, we found no perception of depth in ACRDS with either brief (80 ms) or more sustained (up to 400 ms) presentations.

The presence of a correlated surround (or at least the absence of an anti-correlated surround) has been found to be critical for disparity discrimination in previous studies. Tanabe et al. [19] argue that this surround allows the discrimination of depth by providing a clear reference against which to judge the disparity of the anti-correlated target. Since anti-correlated stimuli provide only a weak depth signal, judging the relative disparity of two such signals may not provide sufficiently reliable disparity information to allow for accurate depth judgments. Moreover, if disparity is encoded in ACRDS by second-order mechanisms, they may only allow for crude near/far judgements, and not for relative depth judgements between two anti-correlated regions [39]. Consistent with this, Tanabe et al. showed that fine cyclopean shape judgements are not possible in ACRDS [19]. This explanation of the importance of a correlated surround depends on the availability of a reference against which to judge the disparity provided by the anti-correlated target, rather than the processing of this disparity information itself.

**Figure 7:**
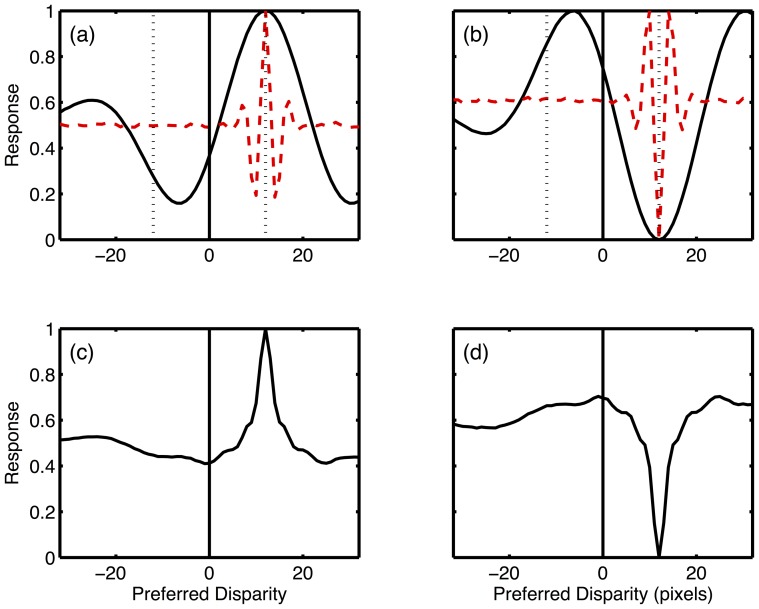
Binocular energy model responses to CRDS and ACRDS. (a) Mean responses of a population of position-tuned neurons to CRDS. Results are plotted as a function of the preferred disparity of each neuron, and show the normalised mean response across 100 stimuli, all with a disparity of 12 pixels. The solid black line shows the response of model neurons tuned to a low frequency; the dotted red line the responses of model neurons tuned to a high frequency. The solid vertical line marks zero disparity and the dotted vertical lines show ± the magnitude of the stimulus disparity. The peak response occurs for neurons tuned to the correct disparity for both frequencies. (b) Shows the responses in the same way for ACRDS. In this case, the peaks occur at different disparities (with different signs) for the two frequencies. (c) Responses to CRDS averaged across four frequencies show a clear peak at the correct disparity (d) For ACRDS, the responses averaged across frequency shows a clear minimum at the correct disparity, but no pronounced peak.

The reversed depth reported in some studies with ACRDS has been linked to the inversion of the disparity tuning functions of binocular neurons for these types of stimuli. This inversion means that the peaks in the response for a given neuron will occur at different disparities from those eliciting the greatest responses for correlated stimuli. Across a population of such neurons, correlated and anti-correlated RDS will elicit the strongest response from neurons tuned to different disparities. However, whether or not this would predict a reversal in depth depends on (i) the disparity of the stimulus (ii) the spatial frequency tuning of the neurons and (iii) how disparity is estimated from the population response. This is shown in figure 7, which presents the results of an implementation of the energy model.

Binocular energy responses were calculated from model neurons with two-dimensional receptive fields given by:
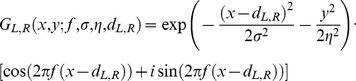
(1)where *x,y* specify the horizontal and vertical location, *f* is the preferred spatial frequency, σ and η specify the envelope of the receptive field, 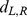 determines the disparity tuning, and L and R refer to the left and right eye's receptive fields. In all cases, receptive fields were identical in shape for the two eyes. The preferred orientation was vertical. Disparity tuning was introduced by shifting the receptive field by equal but opposite amounts (shifts of ±*d*) in the two eyes. Preferred spatial frequencies of 0.025, 0.05, 0.1 and 0.2 cycles/pixel were used. The receptive field envelopes were set to 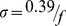 and 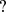. Populations of model neurons with disparity tunings between ±32 pixels were created. The binocular energy response was calculated by convolving the image *I(x,y)* with the left and right receptive fields:
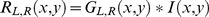
(2)and the binocular energy response was then calculated as:
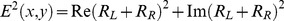
(3)where 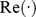 and 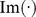 are the real and imaginary components of the complex responses. Binocular energy responses to CRDS and ACRDS were calculated. The stimuli consisted of 513×513 images in which half of the pixels were grey, with a luminance specified as 0. 25% of the pixels, selected randomly, were bright (with a luminance of 1) and another 25%, again selected at random, were dark (with a luminance of -1). For CRDS, the right eye's image was identical to the left eye's, except that is was shifted by 12 pixels horizontally. For ACRDS, the polarity of bright and dark pixels was in addition inverted. The energy response of the population of model neurons to each image was calculated. 100 sample images were generated, and the results are the mean across these samples.

Figure 7a shows the mean responses of two populations of modelled energy neurons to CRDS. Mean responses are normalised so that the maximum for each frequency is 1. The two populations are tuned to two different spatial frequencies, and both show a peak in the population responses for neurons tuned to the correct disparity. Figure 7b shows the responses of the same model neurons, this time to ACRDS stimuli. The population response profile is inverted, meaning that neurons tuned to the correct disparity now produce the smallest response. Peaks occur at the disparities producing the smallest responses for CRDS. These peaks occur at different locations for the two frequencies. In this example, the peak with the smallest magnitude of disparity for the low spatial frequency model neurons occurs for a negative disparity. In contrast, both peaks for the high frequency neuron occur for a positive disparity. It is therefore not clear which sign of perceived depth would be predicted from the energy model. This is illustrated in figures 7c and 7d, which show responses summed over a range of spatial frequencies [3]. In this case, responses were individually normalised (to have a peak of one) before summation. While for CRDS this produces a clear peak at the correct disparity, no such peak is evident for ACRDS. Rather, the only clear feature is a minimum at the stimulus disparity. These results illustrate that, when we consider a population of model energy neurons, tuned to a range of spatial frequencies, we do not, in general, expect ACRDS to signal a clear reversal of depth. Thus, the lack of clear apparent depth for ACRDS that we find is consistent with the incoherent disparity responses that are predicted across a population of disparity energy neurons.
